# Positive relationships and academic stressors in the post-pandemic context of covid-19 in adolescents from a school in Córdoba, Colombia.

**DOI:** 10.1192/j.eurpsy.2023.501

**Published:** 2023-07-19

**Authors:** E. P. Ruiz Gonzalez, M. F. Martinez Burgos, V. Contrera Montiel, M. N. Muñoz Argel, J. J. Diaz Muñoz

**Affiliations:** ^1^ Universidad Pontifica Bolivariana; ^2^Fundacion sociedad, ambiente, emprendimiento y ciclos de vida, Montería, Colombia

## Abstract

**Introduction:**

Berscheid (1999), taken from Lacunza & Contini (2016), indicated that social relations were the foundation of the human condition. From positive psychology, Park et al. (2013) point out good relationships as a factor that contributes to a good psychological life, since they provide emotional and instrumental support in times of stress and challenge, indicating, in turn, normal evolutionary development and the avoidance of psychopathological problems.

**Objectives:**

To establish the relationship between positive relationships and the presence of stressors in adolescents.

**Methods:**

A cross-sectional, descriptive-correlational study was carried out in 109 (N= 109) adolescents. The SISCO Inventory was used to study academic stress as well as the Ryff Psychological Well-Being Scale.

**Results:**

A negative magnitude correlation was found between positive relationships and stressors. ( Table 1).

As a secondary result, 60.6% of the evaluated adolescents presented life purpos as the factor with the highest score in the psychological well-being variable. This points to authors such as Erikson (1988, p. 96), who define adolescence as a space characterized by feelings of creativity, productivity, new ideas, and a period of cognitive and social maturation, which leads to a definitive commitment to life itself. (Graph 1).

**Image:**

**Figure:**
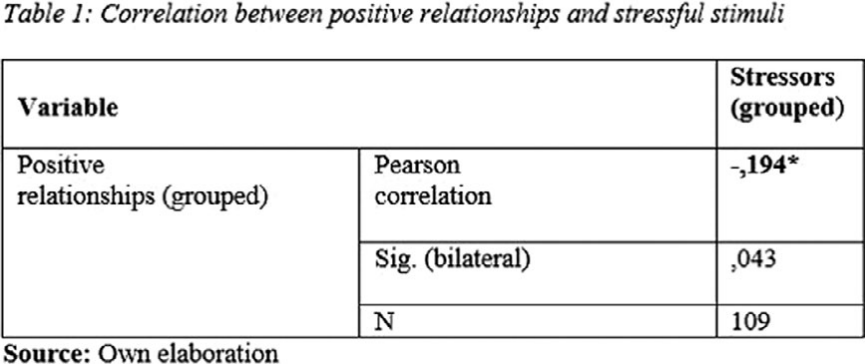

**Image 2:**

**Figure:**
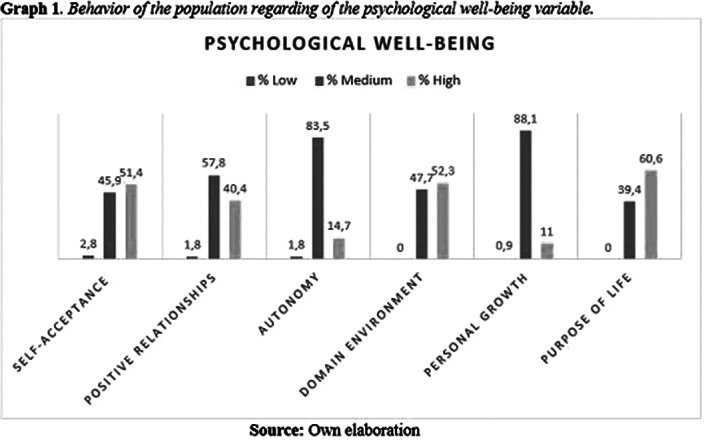

**Conclusions:**

Positive relationships in adolescents decrease the presence of stimuli considered stressors.

**Disclosure of Interest:**

None Declared

